# Correction: UK clinical guideline for the prevention and treatment of osteoporosis

**DOI:** 10.1007/s11657-022-01115-8

**Published:** 2022-05-19

**Authors:** Celia L. Gregson, David J. Armstrong, Jean Bowden, Cyrus Cooper, John Edwards, Neil J. L. Gittoes, Nicholas Harvey, John Kanis, Sarah Leyland, Rebecca Low, Eugene McCloskey, Katie Moss, Jane Parker, Zoe Paskins, Kenneth Poole, David M. Reid, Mike Stone, Julia Thomson, Nic Vine, Juliet Compston

**Affiliations:** 1 Musculoskeletal Research Unit, Bristol Medical School, Learning and Research Building, University of Bristol, Southmead Hospital, Bristol, BS10 5NB UK; 2 Royal United Hospital NHS Foundation Trust, Bath, UK; 3 Western Health and Social Care Trust (NI), Nutrition Innovation Centre for Food and Health, Ulster University, and Visiting Professor, Belfast, Northern Ireland; 4 MRC Lifecourse Epidemiology Centre, University of Southampton, Southampton, UK; 5 NIHR Southampton Biomedical Research Centre, University of Southampton and University Hospital Southampton NHS Foundation Trust, Southampton, UK; 6 NIHR Oxford Biomedical Research Centre, University of Oxford, Oxford, UK; 7 Primary Care Centre Versus Arthritis, School of Medicine, Keele University, Staffordshire, and Wolstanton Medical Centre, Newcastle under Lyme, UK; 8 Centre for Endocrinology, Diabetes and Metabolism, Queen Elizabeth Hospital, University Hospitals Birmingham & University of Birmingham, Birmingham, UK; 9 Mary McKillop Institute for Health Research, Australian Catholic University, Melbourne, Australia and Centre for Metabolic Bone Diseases, University of Sheffield, Sheffield, UK; 10 Royal Osteoporosis Society, Bath, UK; 11 Abingdon and Specialty Doctor in Metabolic Bone Disease, Marcham Road Health Centre, Nuffield Orthopaedic Centre, Oxford, UK; 12 Department of Oncology & Metabolism, MRC Versus Arthritis Centre for Integrated Research in Musculoskeletal Ageing (CIMA), Mellanby Centre for Musculoskeletal Research, University of Sheffield, Sheffield, UK; 13 St George’s University Hospital, London, UK; 14 School of Medicine, Keele University, Haywood Academic Rheumatology Centre, Haywood Hospital, Midlands Partnership NHS Foundation Trust, KeeleStoke-on-Trent, UK; 15 Department of Medicine, University of Cambridge, Cambridge, UK; 16 University of Aberdeen, Aberdeen, Scotland; 17 University Hospital Llandough, Cardiff and Vale University Health Board, Llandough, UK; 18 School of Clinical Medicine, University of Cambridge, Cambridge, UK

**Correction: Archives of Osteoporosis (2022) 17:1–46**

**https://doi.org/10.1007/s11657-022-01061-5**

In this article, the affiliation ‘NIHR Cambridge Biomedical Research Centre’ for author Kenneth Poole was missing.

The original article has been corrected.

